# Detection of specific gene rearrangements by fluorescence *in situ* hybridization in 16 cases of clear cell sarcoma of soft tissue and 6 cases of clear cell sarcoma-like gastrointestinal tumor

**DOI:** 10.1186/s13000-018-0752-6

**Published:** 2018-09-15

**Authors:** Keiko Segawa, Shintaro Sugita, Tomoyuki Aoyama, Terufumi Kubo, Hiroko Asanuma, Taro Sugawara, Yumika Ito, Mitsuhiro Tsujiwaki, Hiromi Fujita, Makoto Emori, Tadashi Hasegawa

**Affiliations:** 1 Department of Surgical Pathology, Sapporo Medical University, School of Medicine, Sapporo, Hokkaido 060-8543 Japan; 2 Department of Pathology, Sapporo Medical University, School of Medicine, Sapporo, Hokkaido 060-8556 Japan; 3 Department of Orthopedic Surgery, Sapporo Medical University, School of Medicine, Sapporo, Hokkaido 060-8543 Japan

**Keywords:** Clear cell sarcoma of soft tissue (CCSST), Clear cell sarcoma-like gastrointestinal tumor (CCSLGT), *EWSR1-ATF1*, *EWSR1-CREB1*, *EWSR1-CREM*

## Abstract

**Background:**

Clear cell sarcoma of soft tissue (CCSST) and clear cell sarcoma-like gastrointestinal tumor (CCSLGT) are malignant mesenchymal tumors that share some pathological features, but they also have several different characteristics. They are well known to express chimeric fusions of Ewing sarcoma breakpoint region 1 (*EWSR1*) and cAMP response element-binding protein (*CREB*) family members; namely, *EWSR1-*activating transcription factor 1 (*ATF1*) and *EWSR1-CREB1*. In addition, recent studies have suggested the presence of other fusions.

**Methods:**

We used fluorescence *in situ* hybridization to detect specific rearrangements including *EWSR1*, *ATF1*, *CREB1*, and cAMP response element modulator (*CREM*) in 16 CCSST and 6 CCSLGT cases. We also used reverse transcription polymerase chain reaction (RT-PCR) to detect specific chimeric fusions of *EWSR1-ATF1* and *EWSR1-CREB1* using fresh tumor samples in available cases.

**Results:**

A total of 15 of 16 CCSST cases (93.8%) had *EWSR1* rearrangement, of which 11 (68.8%) also had *ATF1* rearrangement, suggestive of the presence of *EWSR1-ATF1* fusions. One CCSST case (6.3%) was found to have *EWSR1* and *CREM* rearrangements, and 4 of 6 CCSLGT cases (66.7%) had *EWSR1* rearrangement, of which 2 (33.3%) showed *ATF1* rearrangement and the other 2 cases (33.3%) showed *CREB1* rearrangement. These cases most likely had *EWSR1-ATF1* and *EWSR1-CREB1* fusions, respectively. RT-PCR was performed in 8 available cases, including 6 CCSSTs and 2 CCSLGTs. All CCSSTs showed *EWSR1-ATF1* fusions. Among the 2 CCSLGT cases, one had *EWSR1-ATF1* fusion and the other had *EWSR1-CREB1* fusion.

**Conclusions:**

Rearrangements of *EWSR1* and *ATF1* or *EWSR1-ATF1* fusion were predominantly found in CCSST, whereas those of *EWSR1* and *CREB1* or *EWSR1-CREB1* tended to be detected in CCSLGT. A novel *CREM* fusion was also detected in a few cases of CCSST and CCSLGT. The cases in which *EWSR1* rearrangement was detected without definitive partner genes should be considered for the presence of *CREM* rearrangement.

## Background

Clear cell sarcoma of soft tissue (CCSST) is a malignant mesenchymal tumor that mostly affects young adults and tends to affect the lower extremities, close to the tendon and aponeuroses [1]. Histologically, CCSSTs have epithelioid tumor nests accompanied by some spindling areas, and wreath-like multinucleated giant cells. CCSSTs present with a melanocytic differentiation and often express melanocytic markers including S-100 protein, melanoma antigen (Melan-A), human melanoma black 45 (HMB45), microphthalmia-associated transcription factor (MITF), and SRY-Box 10 (SOX-10) on immunohistochemistry (IHC). Ultrastructurally, CCSST has premelanosomes in the cytoplasm of tumor cells and shares some characteristic features with malignant melanomas (MMs). MMs genetically have BRAF mutations, although CCSST lacks this mutation. Clear cell sarcoma-like gastrointestinal tumor (CCSLGT) is also a malignant mesenchymal tumor that shares some pathological features with CCSST and arises from the gastrointestinal tract, such as the small and large intestine, and stomach. CCSLGT was originally reported to be an “osteoclast-rich tumor of the gastrointestinal tract with features resembling clear cell sarcoma of the soft parts” [2] and the first case of CCSLGT was reported by Alpers et al. [3] as a “malignant neuroendocrine tumor of the jejunum with osteoclast-like giant cells” in 1985. Subsequently, the term CCSLGT was first used by Kosemehmetoglu et al. [4] in their review, which included 13 CCSLGT cases. However, some authors have proposed using the term “malignant gastrointestinal neuroectodermal tumor,” because CCSLGTs lack melanocytic differentiation on IHC and ultrastructural examination and appear to have poorer prognosis [5]. Although CCSLGT has a similar histology to CCSST in some respects, such as a clear cytoplasm and epithelioid cells, there are some differing characteristics. CCSLGT has a pseudo-papillary growth pattern and many osteoclast-type giant cells, and the tumor cells tend to be positive for S-100 protein but show less expression of melanocytic markers on IHC [6]. Genetically, CCSST and CCSLGT usually have characteristic chimeric fusions of Ewing sarcoma breakpoint region 1 (*EWSR1*) with cAMP response element-binding protein (*CREB*) gene family members, *EWSR1-*activating transcription factor 1 (*ATF1*) and *EWSR1-CREB1*, which were derived from each translocation of t(12;22)(q13;q12) and t(2;22)(q34;q12), respectively [7–10]. *EWSR1-ATF1* fusion is much more frequent than *EWSR1-CREB1* fusion, but *EWSR1-CREB1* fusion of CCSLGT is comparatively often observed.

In this study, we used fluorescence *in situ* hybridization (FISH) and reverse transcription polymerase chain reaction (RT-PCR) to perform genetic analyses of 22 cases of CCSSTs and CCSLGTs, and compared their different chimeric fusion types.

## Methods

### Case selection

The study protocol for the collection of tumor samples and clinical information were approved by the Institutional Review Board of Sapporo Medical University Hospital (Sapporo, Japan; No. 292–3012). We selected 22 cases of clear cell sarcoma (CCS) including 16 CCSST and 6 CCSLGT cases from the clinicopathological archive at the Department of Surgical Pathology, Sapporo Medical University Hospital. We reviewed all hematoxylin and eosin-stained sections and confirmed that each case fulfilled the histologic criteria of CCSST and CCSLGT.

### Immunohistochemistry

We evaluated previously reported IHC findings of melanocytic markers, including S-100 protein, Melan-A, HMB45, and SOX-10, and assessed their positivity. We also performed additional IHC using representative sections from formalin-fixed and paraffin-embedded tissues in some cases. These tissues were sliced into 3-mm-thick sections and examined with an automated IHC system at Sapporo Medical University Hospital. All slides were loaded into a PT Link module (Agilent Technologies, Santa Clara, CA) and subjected to a heat-induced antigen-retrieval protocol with EnVision FLEX Target Retrieval Solution (Agilent Technologies) before being transferred to the Autostainer Link 48 instrument (Agilent Technologies) and Dako Omnis (Agilent Technologies). We used antibodies against the following antigens: S-100 protein (polyclonal; Agilent Technologies), Melan-A (A103; Agilent Technologies), HMB45 (HMB45; Agilent Technologies), and SOX-10 (N-20; Santa Cruz Biotechnology, Santa Cruz, CA).

### Fluorescence *in situ* hybridization

We performed FISH using the specimens obtained from tumor materials and 4 μm slices on glass slides. First, we selected an area showing representative histology and marked a 5-mm-diameter circle with a marker on the glass slides for FISH analyses. We performed FISH using dual color break apart probe for EWSR1 (Abbott, Abbott Park, IL), ATF1 (Empire Genomics, Buffalo, NY), CREB1 (Empire Genomics), and CREM (Empire Genomics). FISH was conducted as previously described [11], with the following modifications: baking (1 h at 60 °C), deparaffinization, target gene activation (20 min with 0.2 M HCl followed by 30 min with pretreatment solution at 80 °C), enzyme treatment (60 min with protease solution at 37 °C), re-fixation (10 min in 10% formalin neutral buffer solution), denaturation (5 min with denaturation solution at 72 °C), washing and dehydration (1 min each in 70%, 85%, and 100% ethanol), hybridization with 10 mL DNA probe solution (5 min at 90 °C followed by 48 h at 37 °C), and washing with post-hybridization wash buffer (2 min at 72 °C). As a counterstain, 10 μL 4,6-diamidino-2-phenylindole was added. Slides were coverslipped for viewing under a fluorescence microscope.

To detect the presence of *EWSR1*, *ATF1, CREB1*, and *CREM* rearrangements, we counted 50 nuclei in tumor cells that showed a pair of fused and split signals, and calculated the percentage of split signals. The signals were considered split when the distance between the red and green signals was at least twice the estimated signal diameter. We did not evaluate any truncated and overlapping cells in FISH analysis. We considered the specimen to be “split positive” if split signals were observed in more than 10% of tumor cells [12].

### Reverse transcription-polymerase chain reaction

We detected chimeric fusions by RT-PCR using fresh tumor samples in several available cases. RT-PCR analysis was performed for *EWSR1-ATF1* and *EWSR1-CREB1* fusions. For RT-PCR detection of the *EWSR1-ATF1* and *EWSR1-CREB1* fusions, we used the forward primer EWSex7-F1 with either the CREB1ex7-RevA primer (binds both *CREB1* and *ATF1*; sequence: TCCATCAGTGGTCTGTGCATACTG) or the CREB1ex7-RevC primer (specific for *CREB1*; sequence: GTACCCCATCGGTACCATTGT) [1, 7, 13].

## Results

### Clinical findings

This study involved 8 male and 14 female patients with a mean age of 40 years (range, 8–78 years). Mean tumor size was 4.6 cm (range, 2–10). The anatomical locations were deep soft tissue of the upper (*n* = 6) and lower (*n* = 8) extremities, esophagus (*n* = 1), small intestine (*n* = 5), abdominal wall (n = 1), and skin (n = 1). The primary site in Case 13 was the upper extremity, but a specimen was not available, so we used lymph node specimens of metastatic lesions. Mean follow-up duration was 38 months (range, 3–249 months; Table 1).

**Table 1:** Summary of clinical, immunohistochemical, and genetic findings of CCSST and CCSLGT cases

No.	Age (years) /Sex	Location	Tumor size (cm)	Outcome (months)	Immunohistochemistry			Fluorescence in situ hybridization (%)				Expected fusion genes by FISH	RT-PCR findings
					S-100 protein	Melan-A and/or HMB45	SOX-10	EWSR1	ATF1	CREB1	CREM		
1	29/F	Leg	5	DOD (73)	+	+	NP	72	48	2	NP	*EWSR1-ATF1*	NP
2	41/M	Leg	4	DOD (62)	+	+	NP	68	ND	ND	NP	*EWSR1*- (unknown partner)	NP
3	25/M	Leg	8	DOD (29)	+	+	NP	50	32	4	NP	*EWSR1-ATF1*	*EWSR1* exon 8-*ATF1* exon 4
4	62/F	Leg	2	NED (6)	+	+	NP	8	6	0	NP	Unknown	*EWSR1* exon 8-*ATF1* exon 4
5	33/M	Leg	2.5	DOD (8)	+	+	NP	16	0	0	NP	*EWSR1*- (unknown partner)	*EWSR1* exon 10-*ATF1* exon 5
6	34/F	Leg	NA	AWD (16)	+	+	NP	62	32	0	NP	*EWSR1-ATF1*	NP
7	34/M	Leg	6	AWD (9)	+	+	NP	76	66	6	NP	*EWSR1-ATF1*	NP
8	12/F	Leg	2	NED (96)	+	–	–	36	0	2	2	*EWSR1*-(unknown partner)	NP
9	39/F	Arm	3	DOD (10)	+	+	NP	74	54	ND	NP	*EWSR1-ATF1*	NP
10	69/F	Arm	2	DOD (26)	+	+	NP	50	58	0	NP	*EWSR1-ATF1*	*EWSR1* exon 8-*ATF1* exon 4
11	41/M	Arm	2.5	AWD (11)	+	+	NP	58	58	2	NP	*EWSR1-ATF1*	*EWSR1* exon 10-*ATF1* exon 5
12	40/M	Arm	5.5	AWD (23)	+	+	NP	70	36	8	NP	*EWSR1-ATF1*	*EWSR1* exon 8-*ATF1* exon 5
13	49/F	Arm	4	AWD (24)	+	+	NP	70	8	4	36	*EWSR1-CREM*	*
14	56/F	Arm	3.9	AWD (3)	+	+	NP	80	86	2	0	*EWSR1-ATF1*	NP
15	8/F	Abdominal wall	9.5	DOD (249)	+	+	NP	50	36	0	NP	*EWSR1-ATF1*	NP
16	43/M	Skin	3	NED (29)	+	–	NP	84	76	0	NP	*EWSR1-ATF1*	NP
17	41/F	Ileum	4	NED (28)	+	–	NP	72	58	2	NP	*EWSR1-ATF1*	NP
18	78/F	Ileum	9	NED (48)	+	–	+	2	2	0	12	(unknown partner)-*CREM*	NP
19	38/F	Small intestine	10	DOD (17)	+	–	NP	56	6	42	NP	*EWSR1-CREB1*	NP
20	20/F	Ileum	4	NED (7)	+	–	+	80	94	4	NP	*EWSR1-ATF1*	*EWSR1* exon 8-*ATF1* exon 4
21	47/F	Ileum	4.5	AWD (34)	+	–	+	74	0	62	NP	*EWSR1-CREB1*	*EWSR1* exon 7-*CREB1* exon 7
22	57/M	Esophagus	3	NED (18)	+	–	+	2	2	0	8	Unknown	NP

*+* Positive, − Negative, *AWD* Alive with disease, *NED* No evidence of disease, *DOD* Died of disease, *NA* Not available, *ND* Not detected, *NP* Not performed, *FISH* Fluorescence in situ hybridization, *RT-PCR* Reverse transcription PCR; *, fusion gene was not detected by RT-PCR in formalin-fixed, paraffin-embedded materials

### Histological and IHC findings

**Fig. 1:**
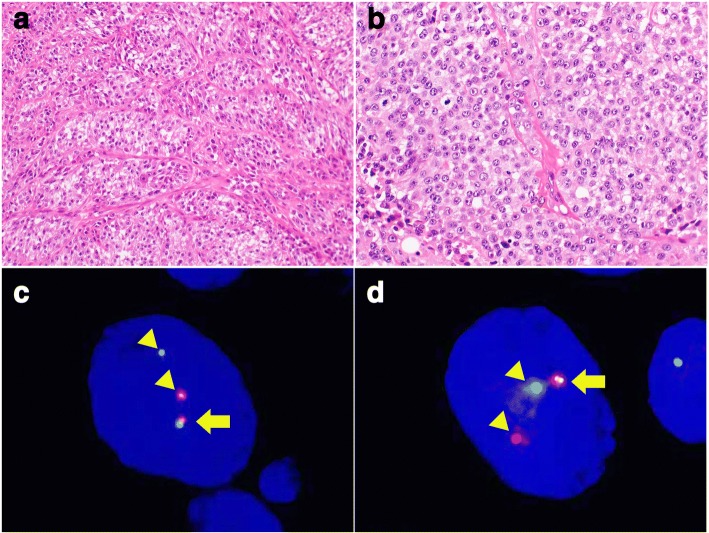
Pathological findings of CCSST with *EWSR1* and *ATF1* rearrangements. **a** CCSSTs showed sheet-like and nested growth patterns of polyhedral tumor cells (200×). **b** Tumor cells had round nuclei with conspicuous nucleoli and clear to pale eosinophilic cytoplasm (400×). **c** FISH of *EWSR1* split signals. Tumor cells showed *EWSR1* split signal with a pair of fused (arrow) and split (arrow head) patterns (1000×). **d** FISH of *ATF1* split signals. Tumor cells showed *EWSR1* split signal with a pair of fused (arrow) and split (arrow head) patterns (1000×)

**Fig. 2:**
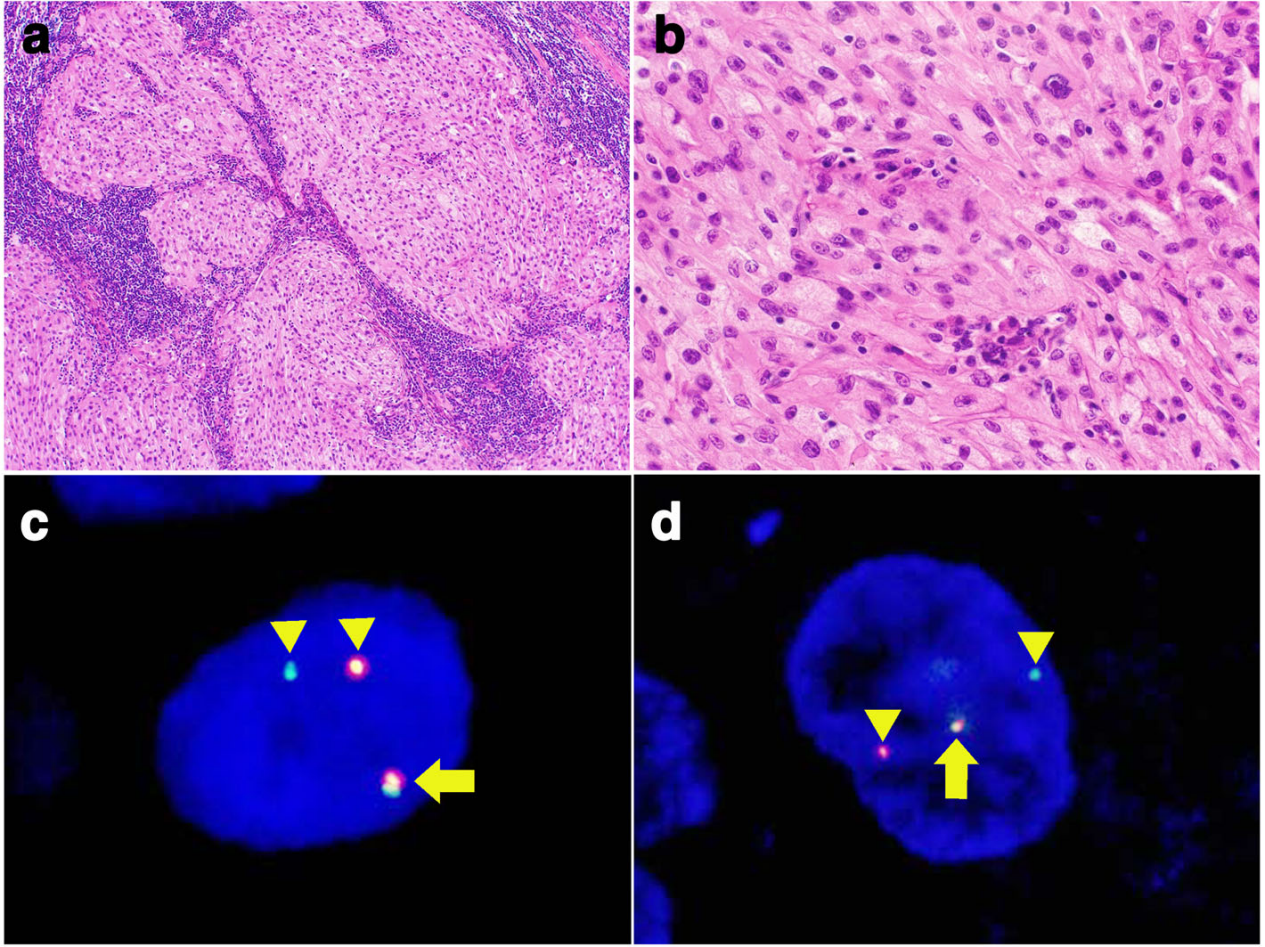
Pathological findings of CCSST with *EWSR1* and *CREM* rearrangements. **a** Metastatic CCSST of the lymph nodes (100×). **b** Tumor cells were polyhedral to spindle-shaped and had oval to round nuclei with pale to clear and eosinophilic cytoplasm (400×). **c** FISH of *EWSR1* split signals. Tumor cells showed *EWSR1* split signal with a pair of fused (arrow) and split (arrow head) patterns (1000×). **d** FISH of *CREM* split signals. Tumor cells showed *CREM* split signal with a pair of fused (arrow) and split (arrow head) patterns (1000×)

**Fig. 3:**
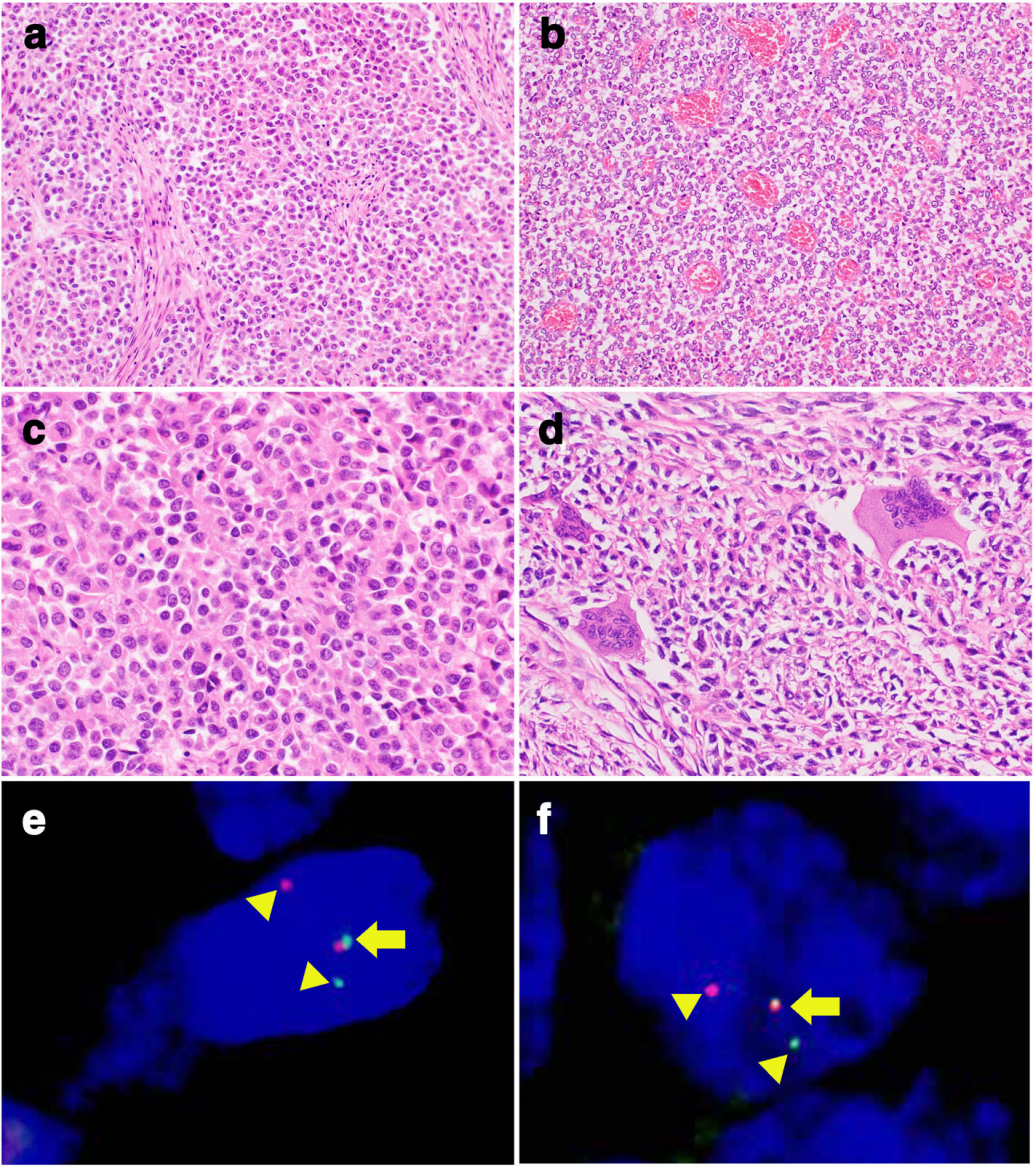
Pathological findings of CCSLGT with *EWSR1* and *CREB1* rearrangements. **a** CCSLGTs often exhibited sheet-like proliferation of polyhedral and epithelioid tumor cells (200×). **b** Areas of pseudo-papillary growth pattern around the vasculatures were also observed (200×). **c** Tumor cells had mildly irregular-shaped round nuclei showing a coarse chromatin pattern and lightly eosinophilic to less frequently clear cytoplasm. Nucleoli were inconspicuous (400×). **d** Osteoclast-type giant cells were scattered in the tumor (400×). **e**. FISH of *EWSR1* split signals. Tumor cells showed *EWSR1* split signal with a pair of fused (arrow) and split (arrow head) patterns (1000×). **f**. FISH of *CREB1* split signals. Tumor cells showed *CREB1* split signal with a pair of fused (arrow) and split (arrow head) patterns (1000×)

Histologically, the majority of CCSSTs showed sheet-like and nested growth patterns of epithelioid and/or spindle tumor cells that had round to mildly irregularly shaped nuclei with conspicuous nucleoli and clear to pale eosinophilic or amphophilic cytoplasm (Fig. 1a, and b). Among the 16 CCSSTs, 13 cases showed predominant epithelioid cytology and 3 cases exhibited predominant spindle cytology. Multinucleated giant cells were sparsely found in 14 cases. Visible melanocytic differentiation (melanin pigmentation) was observed in 4 cases. The predominant architecture was sheet-like and nested in 12 cases, fascicular in 3 cases, and pseudo-papillary in 1 case. Nucleolar prominence was found in 14 cases. Case 13, which had both *EWSR1* and *CREM* rearrangements (metastatic lesions in lymph nodes), showed sheet-like and fascicular proliferation of epithelioid and spindle tumor cells that had round nuclei with conspicuous nucleoli and abundant pale eosinophilic cytoplasm (Fig. 2a, and b); no myxoid change was found. On the other hand, CCSLGTs often exhibited pseudo-papillary patterns of epithelioid tumor cells, with round to irregular-shaped nuclei showing a coarse chromatin pattern and having a slightly eosinophilic to less clear cytoplasm (Fig. 3a, and b). All 6 CCSLGT cases showed predominant epithelioid cytology and did not exhibit visible melanocytic differentiation. The predominant architecture was pseudo-papillary in 3 cases and sheet-like in 3 cases. Nucleoli were less conspicuous than those from CCSSTs (Fig. 3c), and nucleolar prominence was observed in 2 cases. Osteoclast-type giant cells were scattered in 5 cases (Fig. 3d). Case 18, which had *CREM* rearrangement, showed pseudo-papillary proliferation of epithelioid cells that had round nuclei without conspicuous nucleoli and pale eosinophilic cytoplasm. There were no histologic patterns indicating any correlation between *ATF1* and *CREB1* or indicating cases with *CREM* rearrangement.

We performed IHC of melanocytic markers, including S-100 protein, Melan-A, HMB45, and SOX-10, and assessed their positivity (Table 1). In Cases 8 and 16, CCS of the deep soft tissue and skin showed no reactivity with Melan-A and HMB45 and Case 13 with both *EWSR1* and *CREM* rearrangements was negative for Melan-A, but almost all of the CCSST cases were positive for all melanocytic markers. In contrast, CCSLGTs showed no reactivity with any melanocytic markers and were positive for only S-100 and SOX-10. All of the IHC results were compatible with the pathological diagnosis of CCSST and CCSLGT.

### Fluorescence *in situ* hybridization

As shown in Table 1, 15 of the 16 CCSST cases (93.8%) had *EWSR1* rearrangement, of which 11 (68.8%) also showed *ATF1* rearrangement (Fig. 1c, and d), suggestive of the presence of *EWSR1-ATF1* fusion. One CCSST (Case 13) (6.3%) exhibited *EWSR1* and *CREM* rearrangements (Fig. 2c, and d), indicating *EWSR1-CREM* fusion although no fusion gene was proven by RT-PCR using formalin-fixed, paraffin-embedded materials (data not shown). Two CCSSTs with *EWSR1* rearrangement had no partner genes: one (Case 8) showed no rearrangement of *ATF1*, *CREB1*, or *CRE*M, and one (Case 2) did not exhibit any rearrangement. Four of 6 CCSLGT cases (66.7%) exhibited *EWSR1* rearrangement: 2 (33.3%) showed *ATF1* rearrangement and the other 2 (33.3%) showed *CREB1* rearrangement (Fig. 3e, and f), suggestive of the presence of *EWSR1-ATF1* and *EWSR1-CREB1* fusions, respectively. One case (Case 18) was positive for split *CREM* signals, although no partner genes were detected. One case (Case 22) showed no rearrangement of *EWSR1*, *ATF1*, *CREB1*, or *CREM*.

### Reverse transcription-polymerase chain reaction

RT-PCR was performed in 8 available cases including 6 CCSSTs and 2 CCSLGTs (Table 1). All 6 CCSST cases showed *EWSR1-ATF1* fusion between *EWSR1* exon 8 and *ATF1* exon 4, *EWSR1* exon 10 and *ATF1* exon 5, or *EWSR1* exon 8 and *ATF1* exon 5. Although *EWSR1-ATF1* fusion was confirmed by RT-PCR in 2 cases (Cases 4 and 5), adequate *EWSR1* or *ATF1* split signals were not detected by FISH. Among the 2 CCSLGT cases, one had *EWSR1-ATF1* fusion between *EWSR1* exon 8 and *ATF1* exon 4, and the other had *EWSR1-CREB1* fusion between *EWSR1* exon 7 and *CREB1* exon 7.

## Discussion

Although CCSST and CCSLGT share similar pathological findings, there are apparent morphological and immunohistochemical differences between the two tumor types. We confirmed the differences in histology and IHC results in our cohort cases. Histologically, the cytological findings of tumor cells and architectural proliferation pattern differed. The tumor cells of CCSST were polyhedral to epithelioid, and spindle-shaped with round to mildly irregular-shaped nuclei and conspicuous nucleoli. In contrast, the tumor cells of CCSLGT had epithelioid tumor cells with irregular-shaped nuclei showing a coarse chromatin pattern and more eosinophilic cytoplasm. Nucleoli were not remarkable in CCSLGT compared to CCSST. CCSST exhibited sheet-like, solid, and nested tumor cell proliferation. In contrast, CCSLGT additionally showed a pseudo-papillary growth pattern. The existence of scattered osteoclast-type giant cells was also characteristic of CCSLGT. CCSSTs were positive for Melan-A and/or HMB45 melanocytic markers in addition to S-100 protein, as determined by IHC. On the other hand, CCSLGTs were not reactive for any melanocytic markers, with the exception of S-100 protein and SOX-10. As in previously reported studies, *EWSR1-CREB1* fusion tended to be detected in CCSLGTs. This genetic tendency might reflect the morphological and immunohistochemical differences between the two tumor types.

The novel finding of the study was that we discovered *CREM* rearrangement in a few CCSs. Kao et al. [14–16] stated that an *EWSR1-CREM* fusion was previously detected by RNA sequencing in 2 melanoma cell lines (CHL-1 and COLO 699) and proposed that these cell lines may have originated from CCS because of the histological and immunohistochemical overlap between malignant melanoma and CCS. On the other hand, *EWSR1-CREM* fusion was found in a unique myxoid mesenchymal tumor that was recently described as a new entity [14, 17]. This myxoid tumor is thought to have an intracranial location, and 8 cases have previously been reported, of which 7 occurred in intracranial lesions like meninges, brain tumors, and ventricles, and one case arose in the pelvic/perirectal region. A genetic study revealed *EWSR1* fusions with *CREB* family genes in all of these tumors. Among the 8 tumors, 3 had *EWSR1-CREM* fusion, 4 had *EWSR1-CREB1* fusion, and one tumor showed *EWSR1-ATF1* fusion. However, histological and immunohistochemical findings completely differed between this particular myxoid mesenchymal tumor and CCSST/CCSLGT, and interestingly, these genetic results corresponded to those of CCSST and CCSLGT.

CCSLGT was originally reported as an “osteoclast-rich tumor of the gastrointestinal tract with features resembling clear cell sarcoma of the soft parts” [2]. However, some authors prefer to refer to CCSLGT as a “malignant gastrointestinal neuroectodermal tumor” (GNET), because these tumors lack evidence of melanocytic differentiation [5]. A recent review discussed the relationship between clear cell sarcoma of the gastrointestinal tract (CCS-GIT) with GNET. There were differences in morphology and IHC findings between CCS-GIT and GNET. GNET tended to show a wider spectrum of growth patterns, including a pseudo-papillary growth pattern, and exhibited no evidence of melanocytic differentiation. Clinically, CCS-GIT affected males more often than females, and GNET occurred in younger patients although no significant differences existed in their biological behaviors [18]. It has been reported that GNET has poorer prognosis than CCS-GIT [5], but additional studies are needed to clarify the relationship between the two entities.

While a previous study revealed that *EWSR1-ATF1* fusion was identified by RT-PCR in 91% of CCSST cases [1], we detected *EWSR1-ATF1* fusion in 13 of 16 CCSST (81.3%) both by FISH and RT-PCR. Moreover, after excluding 1 case of *EWSR1-CREM* fusion, 13 of 15 CCSST cases (86.7%) had *EWSR1-ATF1* fusion. The percentage of positive cases in the present study nearly reached that of the previous study. CCSLGT has been identified as having *EWSR1-ATF1* or *EWSR1-CREB1* fusion, with CCSLGT more frequently having *EWSR1-CREB1* fusion. These fusions have also been detected in angiomatoid fibrous histiocytoma and primary pulmonary myxoid sarcoma despite the morphological and immunohistochemical differences between CCSST/CCSLGT and these tumors. To detect specific fusions, FISH is an effective and useful tool using formalin-fixed, paraffin-embedded sections in routine pathological work. In this study, we successfully detected *EWSR1*, *ATF1*, *CREB1*, and *CREM* rearrangements by FISH in the majority of cases. Some cases showed only one specific rearrangement and did not reveal any rearrangement of partner genes. In such cases, it is expected that certain unknown partner genes can form some novel chimeric genes, and that powerful analytic tools such as next-generation sequencing will be useful for detecting these novel fusions.

## Conclusions

Rearrangements of *EWSR1* and *ATF1* or *EWSR1-ATF1* fusion were predominantly found in CCSST, whereas those of *EWSR1* and *CREB1* or *EWSR1-CREB1* tended to be detected in CCSLGT. We detected a novel *CREM* rearrangement in surgical CCSST specimens by FISH. Although this novel rearrangement occurred in a minority of CCSST cases, further studies are needed to elucidate its pathological significance.

## Abbreviations

CCS: Clear cell sarcoma
CCSLGT: Clear cell sarcoma-like gastrointestinal tumor
CCSST: Clear cell sarcoma of soft tissue
FISH: Fluorescence *in situ* hybridization
IHC: Immunohistochemistry
